# Vitamin K supplementation during pregnancy for improving outcomes: a systematic review and meta-analysis

**DOI:** 10.1038/s41598-018-29616-y

**Published:** 2018-07-30

**Authors:** Sadequa Shahrook, Erika Ota, Nobutsugu Hanada, Kimi Sawada, Rintaro Mori

**Affiliations:** 1 Department of Health Policy, National Center for Child Health and Development, Tokyo, Japan; 2 Global Health Nursing, St. Luke’s International University, Graduate School of Nursing Sciences, Tokyo, Japan; 3 Department of Food Science and Nutrition Faculty of Human Life and Environmental Sciences, Nagoya Women’s University, Aichi, Japan; 4 Present Address: Population Health Research Institute, A Joint Institute of McMaster University and Hamilton Health Sciences, Hamilton, Ontario Canada

## Abstract

To study supplementation effect of vitamin K (VK) alone or combined with other nutrients administered to pregnant women, we searched Cochrane Pregnancy and Childbirth Group’s Trials Register (till 22 January 2016, updated on 28 February 2018) including other resources. Two review authors independently assessed randomised or quasi-randomised controlled trials for inclusion, data extraction, accuracy, and risk of bias. We included older trials from high-income countries (six; 21,493 women-newborns), judged mostly as high or unclear bias risk. We could not assess high-risk e.g. epileptic women, but healthy women (different gestational ages) received varying VK dosages and duration. We meta-analysed neonatal bleeding (RR 1.16, 95% CI 0.59 to 2.29; *P* = 0.67) and maternal plasma VK1 (MD 2.46, 95% CI 0.98 to 3.93; *P* = 0.001). We found many outcomes were un-assessed e.g. perinatal death, maternal bleeding, healthcare utilization. Mostly newborns were included where VK found significantly effective for e.g. serum VK (mother-newborn), maternal breast milk VK. Few trials reported neonatal adverse side effects. The GRADE evidence quality was very low i.e. neonatal bleeding, neonatal jaundice, maternal plasma VK1. The intervention was favourable for maternal sera VK1 but remained uncertain for neonatal bleeding and other outcomes. The existing literature gaps warrant future investigations on un-assessed or inadequately reported outcomes.

## Introduction

Deficiency of vitamin K (VK) can be critical for pregnant women and especially newborns, possibly resulting in haemorrhage. Prothrombin requires VK for blood coagulation. Therefore, when prothrombin levels drop, blood-clotting also slows down and may result in excessive bleeding in mothers or neonates^1^. Adults can merely suffer from VK deficiency, however, this is still possible in case of an impaired absorption due to a latent aetiology^2,3^. The role of VK during pregnancy is largely unknown^4^. However, since women in pregnancy require higher a nutrient supply, they may experience greater shortages of other nutrients, especially in suboptimal deficiency^5^. Deficiency of VK can worsen when certain drugs such as heparin and carbamazepine are consumed during pregnancy, because the drugs can impede women’s metabolism of VK^1,6^. Additionally, the fetus can be affected from the exposure of the drugs in utero, in which onset of coumarin embryopathy (CE) is possible^7^. About 6% of newborns can develop CE if exposed to maternal coumarin consumption during pregnancy and 80% of these babies can be diagnosed with skeletal abnormalities such as midfacial hypoplasia. Coumarins can impede fetal coagulation by passing into women’s placenta; as a result, newborns (reportedly 10%) who are diagnosed with CE can be at risk of intracranial haemorrhage (IH)^8^. Twenty-two percent of pregnant women taking coumarin anticoagulants experienced miscarriage^8^, although evidence is insufficient supporting a causal link between VK deficiency and miscarriage. Furthermore, nutritional deficits^9^ of some vitamins such as VK, vitamin B_12_ and trace minerals may result in unfavourable pregnancy outcomes in reproductive aged women receiving bariatric surgery^5,10^.

Newborn bleeding disorders require immediate treatment with VK supplements. Vitamin K deficiency bleeding (VKDB) at birth results in haemorrhage inside the newborns’ skull^11^. Newborns’ VK is naturally low at birth and the transfer of maternal VK from the breast milk to the placenta is slow^11^. Deficiency of VK immediately post-birth is very likely in premature babies with existing VK deficits and suboptimal oral absorption^12^. Newborns’ early VKDB occurs immediately at birth or in 24 hours before delivery^13^. Late VKDB incidents without VK prophylaxis occurred in five out of 105 births in Western European countries, and 72 and 11 out of 105 births in Thailand and Japan, respectively^11^. An estimated 0.01% to 0.44% of newborns with VKDB were reported receiving no VK immediately after birth^14^. A 20% mortality was predicted in babies with severe haemorrhage, including 50% of IH and common continuous neurologic impairment^15,16^. Offspring of women with epilepsy (WWE) suffer from unfavourable outcomes, for instance newborns have higher likelihood of experiencing early haemorrhage and neural tube defects from maternal anticonvulsant intake, which obstructs metabolism of phytomenadione (VK) and folic acid^17–19^. Although uncommon, newborns liver disease can be increased, if pregnant women consume VK antagonists^7^.

Administration and dosages of VK are shown to be varying. When VK absorption is impaired, for instance in cystic fibrosis, celiac disease, or other condition, taking VK supplements is required, ideally in multivitamin form, which is considered to be more beneficial than VK alone^4^. Pregnant women consuming anticonvulsant drugs are advised to have VK either 2 or 4 weeks before delivery^4,17^ and should be carefully assessed^20^. From 36 weeks of gestation, women taking anticonvulsants^21^ were given parenteral or oral VK dosages e.g. 10 mg of intramuscular VK daily for two to seven days^22^ and 10 mg of VK1 daily. The over the counter sale of varying forms of VK in the United States (US) is wide, for example: capsules, water-soluble chlorophyll tablets, and liquid^4^. Phylloquinone (VK1) is sold alone as a supplement or as a multivitamin complex in 5 mg tablets^4^. Insufficient evidence exists to show side-effects of excessive VK consumption in pregnant women. However, due to the interactions between VK and some drugs e.g. anticoagulants, VK consumption is regulated for pregnant women and lactating mothers^4^. Since VK crosses into the placenta and is secreted in the breast milk, lactating women should seek advice before starting VK supplements^4^. Oxidative damage, methaemoglobin, and red cell fragility symptoms can be developed during administration of high VK3 (menadione) doses and, if local hypersensitivity reaction is present, mostly occurs from VK1 dermal injections^23^. Experts suggest ≤1 mg is unlikely to cause any harm^24^. When oral VK associated toxicity is still unknown, 10 to 20 mg phylloquinone or above is considered safe for common clinical symptoms in the US; besides, the dosages showed no side effects in patients with chronic fat malabsorption^3^. However, administration of synthetic menadione and its derivatives are restricted, particularly for infants^3^.

Factor VII deficits in megaloblastic anaemia during pregnancy with thrombocytopenia may be improved through antenatal VK therapy^25^. Pregnant women in bariatric surgery can be remedied from severe anaemia, low birthweight (LBW) and congenital abnormalities, if VK and other important micronutrients are adequately taken^5,10^. Neonatal prothrombin and partial thromboplastin activities can be improved through VK administration, including reduced incidence and severity of intraventricular haemorrhage (IVH)^26,27^, although VK had null effect in preventing IVH in a previous review^28^. Antenatal VK may help to reduce haemorrhagic complication risk in infants born to WWE during pregnancy^29,30^, including a lowered occurrence of neonatal VK deficiency^21^. The effects of VK on related deficiency, particularly bleeding complications and other adverse outcomes, including its importance, to date have been predominantly assessed for neonates, but not for reproductive aged women, especially in pregnancy. It is therefore essential to evaluate the efficacy and safety of different VK regimens during pregnancy for improving maternal and newborn outcomes. In this review, including all pregnant women, we assessed the impact of VK supplementation compared with none or VK with other nutrients on maternal and neonatal outcomes. Outcomes assessed in another review^28^ weren’t considered in our investigation.

## Materials and Methods

We developed the present systematic review and meta-analysis following the Cochrane methodology^31^ and PRISMA guidelines^32^.

### Search strategies

The main searches were performed on the Cochrane Pregnancy and Childbirth Group’s (PCG) Trials Register from the inception until 2016 January 22 by a PCG search co-ordinator. The search was populated on major electronic databases: CENTRAL, MEDLINE, Embase, CINAHL, including hand-searches of 30 journals and major conference proceedings, recent awareness alert for 44 additional journals and BioMed Central email alert (http://pregnancy.cochrane.org/pregnancy-and-childbirth-groups-trials-register). Two PCG staff screened the initial results and reviewed full texts of the relevant reports. Before adding to the Register, the intervention detail was used for each report to be assigned with a number related to particular review topics. Using the topic number (not keywords), the Registry search was performed for each review. A precise search set was built using this method and was fully incorporated into this review. We performed an update search (28 February 2018; see Supplementary Information for full search strategies). We also searched bibliographic references of the identified trials, including reports and relevant reviews. We applied neither language nor date restrictions. We contacted researchers in this field for additional information, if needed.

### Study selection

Inclusion of the eligible trials was guided by the independent assessment of two of the review authors (SS, KS or NH). In case of disagreement, the review authors either discussed among the team or consulted an arbitrator (EO or RM). We considered quasi-randomised trials (Cochrane Handbook; Section: 13.2.1.1)^31^ to avoid finding none or a limited number of randomised trials and due to the likely limitations inherited to randomised trial design such as assessment of adverse events that are rare or need long-term observation. We included studies based on the following inclusion criteria: (1) randomised or quasi-randomised controlled trials assessing VK supplementation during pregnancy; (2) trials presented only as abstracts (3) trials that studied pregnant women as a subpopulation and where applicable data could be extracted; (4) regardless of pregnancy stage, all pregnant women and their babies were included; (5) oral, intramuscular, or intravenous VK were administered; (6) outcomes e.g. neonatal bleeding; perinatal mortality; maternal bleeding incidence (primary outcomes); still birth; neonatal death; neonatal bleeding; maternal VK deficiency; anemia; and adverse side effects; and healthcare use e.g. special care/admission to intensive care unit and days of hospitalisation (mother-newborn). See protocol^33^ for full outcome list. We excluded: (1) cross-over trials; (2) trials assessing VK effect in women undergoing imminent preterm delivery to avert newborn PVH (assessed earlier)^28^. Our intervention group was pregnant women who have received antenatal VK only or combined with micronutrients, without restrictions in dose, intake frequency, and length and timing of birth. Pregnant women were compared in three arms: (1) any VK dosage versus none; (2) VK versus placebo or none; (3) micronutrients containing VK versus micronutrients without VK.

### Data extraction and quality assessment

A data extraction form^31^ was developed which was used by two of the review authors (SS, KS or NH). We extracted data as per our protocol^33^ and PICOS specifications including funding source information. We sought discussion or consultation from an arbitrator (EO or RM), if discrepancies occurred. Using Review Manager program^34^, we entered data and validated data accuracy. We contacted authors of the original papers or content experts for clarity regarding pertinent information. We contacted one research group in Japan (identified from included studies) who provided further information towards our data extraction and analysis plan. Using the Cochrane Risk of Bias (RoB) tool^31^, two of the review authors (SS, KS or NH) constructed independent assessments for each of the trial’s methodological quality. We sought discussion or consultation from a third assessor (EO or RM) to resolve disagreement. The trials were assessed in 6 domains: Sequence generation; Concealment of allocation; Blinding of subjects and personnel; Blinding of end point assessment; Incompleteness in outcome data; Selective outcome reporting; Other bias; and Overall bias risk. Following the Cochrane RoB criteria^31^, we developed explicit judgements, whether high, low or unclear bias risk was a concern for each of the trials. For the above domains, we also judged the likely extent and trend of the risk of bias and suggested whether these could affect the findings. Finally, the evidence quality for the following end points was evaluated using the GRADE ranking^35^: perinatal death; newborn bleeding; maternal bleeding; neonatal mortality; preterm birth; neonatal jaundice; maternal plasma VK. We used the GRADEpro program^36^ to import findings from RevMan 5.3 program^34^ and generated ‘Summary of findings’ table. Summary intervention effect and quality evaluation for each outcome was created following the GRADE criteria with five methodological consideration: study limitations, imprecision, indirectness, consistency of effect, and publication bias. The evidence level was put down a level lower from ‘high quality’ since serious (or by two levels for very serious) limitation was found, and was guided by assessment of bias risk, evidence indirectness, serious discrepancy, inaccuracy of effect estimates or probability of publication bias.

### Data synthesis and statistical analysis

Review Manager software^34^ was used to carry out statistical analysis. We meta-analysed data for neonatal bleeding and maternal plasma VK1. For the rest of the outcomes, we either generated effect estimates as per our protocol^33^ or followed a structured data synthesis approach, and presented them in a consistent manner as suggested^31^. We used Tau^2^, I^2^ and Chi^2^ statistics to assess statistical heterogeneity in each meta-analysis and heterogeneity was regarded substantial if an I^2^ reached above 30% and either a Tau^2^ went above zero, or the Chi^2^ test showed P value of <0.10 which is low^31^. Using the GRADE criteria, we assessed the quality of the evidence (as high, moderate, low, very low)^35^ by examining the design and execution of the clinical trials, evidence indirectness, and some additional domains through GRADEpro software. Dichotomous data findings were presented as summary risk ratio and 95% confidence intervals. For continuous data, we presented findings with mean ± standard deviation (SD) as reported by the study authors, or mean difference. If possible, we analysed cluster-randomised trials, heterogeneity and sensitivity analysis, including subgroup analyses and sensitivity analyses on the pooled data to investigate effects of bias risk, multi-arm trials, and publication bias, as recommended^31^. We addressed missing data in the included trials through levels of attrition. If possible, we performed a sensitivity analysis to assess whether large proportions of missing data in the included trials had any impact on the overall evaluation of the intervention. Wherever possible, we analysed outcomes adopting an intention-to-treat approach, i.e. all randomised participants in each arm were included in the analyses, and despite whether the allocated intervention was received or not, all participants were analysed based on their corresponding group of allocation. We derived the denominator of each end point in individual trial by the count of pregnant women randomised minus any women whose end points were identified as missing.

### Data availability

All data in this review (and its Supplementary Information document) are available for public use without any restriction.

## Results

**Figure 1:**
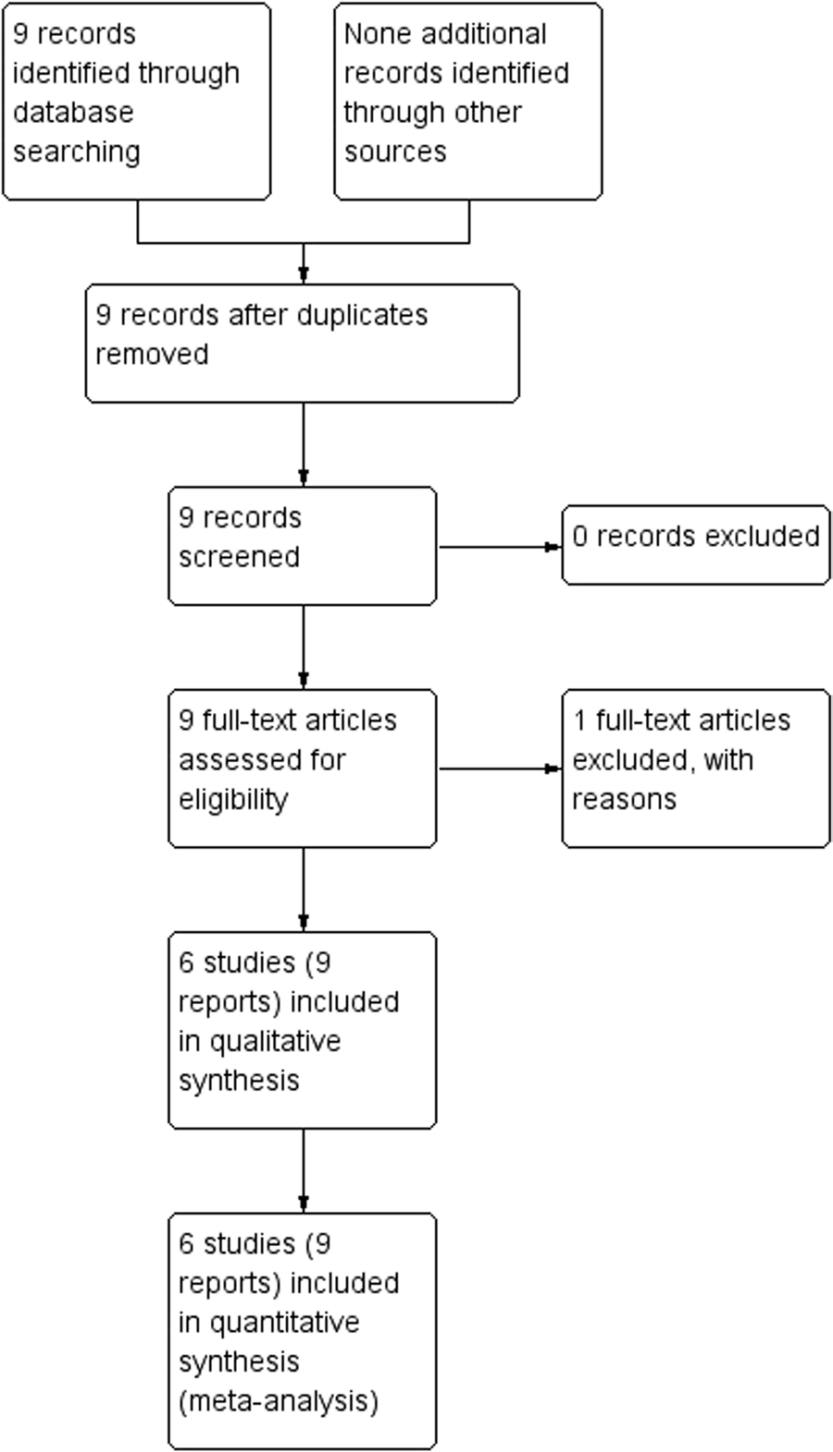
PRISMA study flow identifying potential studies to include in the review on the effect of vitamin K supplementation during pregnancy.

Our search in the Cochrane PCG Trials Register identified nine papers from seven studies, of which 6 (three RCTs, 3 quasi-randomised trials) met our inclusion criteria^36–41^ (Fig. 1). By including pregnant women, the trials assessed VK supplementation effect in comparison to no VK or placebo. The trials were published between 1951 and 1993. All trials were tertiary hospital based and from high-income countries: the US, the United Kingdom (UK), the Netherlands, and Japan^36–41^, except one from South Africa^42^. The South African trial^42^ was excluded because pregnant women in the study groups were not stratified by mature/premature cases. We included 21,493 participants with varying gestational ages: from 34th gestational week^41^ to labour^37,38^, the majority of which were healthy and without a medication use history^36,39–41^. Trials reported oral VK1 with varying dosages and duration e.g. 1 mg/d until delivery and four treatment weeks^41^ and intramuscular VK 50 mg between 4 and 12 hours before delivery^37^. Two each of the trials assessed oral or intramuscular VK and compared with no VK, placebo, or no VK shots. Neonatal bleeding (1/3 review primary outcomes) and surrogate VK deficiency markers e.g. serum Osteocalcin (irOC) and hydroxylapatite binding capacity (HBC), plasma VK1, VK2 and protein-induced VK-absence (PIVKA-II) were assessed (added as non-prespecified outcomes). None of the trials investigated the combined effect of multi-vitamins. See Table 1.

**Table 1:** Characteristics of the included studies.

Study/Design	Inclusion criteria	Exclusion criteria	Participants		Intervention			Comparator	Funding/Conflicts of Interest
			N	Int./Cont.	Route	Time	Vitamin/Dose		
Anai 1993 Oita, Japan/Quasi-RCT	Healthy women w/t drug use, singleton pregnancies, 34–35 gestational wks. on last menstrual period and ultrasound	Women (n = 22) given birth during antenatal VK1 or <10 days after the int.	291	74/186	Oral	2 weeks, minimum 10 days prior delivery	VK1/10 mg daily	No VK	Not specified
Hay 1951 Liverpool, UK/Quasi-RCT	Not specified	Not specified	Some 20,000	4,602/12,136	Intramuscular injection	4 and 12 hours prior delivery	VK/50 mg	No VK shots	Not specified
Hill 1961 Texas, US/Quasi-RCT	Not specified	Not specified	533	266/267	Intramuscular injection	During labour	VK (Hykinone) 2 ampoules/2.5 mg each	Normal saline	Not specified
Kon-Siong 1992 Maastricht, the Netherlands/RCT	Healthy women at 34th gestational wks., w/t drug use, uneventful pregnancy	Not specified	60	24/20	Oral	6 wks. prior delivery	VK1 (Konakinon)/1 mg daily	Placebo	Not specified
Motohara 1990 Kumamoto, Japan/RCT	Regardless of pregnancy stage, all women w/t alcohol and drug use, uneventful pregnancy	Not specified	33	11, 12/10	Oral	7–10 consecutive mornings prior delivery	VK1/20 mg daily	No VK	“…supported by a research grant from the Ministry of Health and Welfare of Japan….”/Unspecified
Owen 1967 Iowa, US/RCT	Specified as “medically indigent women with uncomplicated pregnancies”	Specified as “women whose infant was administered VK after birth were excluded”	Some 500 women	Different samples used for groups and 2 different years	Oral	Final pregnancy week	VK1/5 mg daily	Placebo	Not specified

Abbreviations: RCT = randomised controlled trial; w/t = without; VK = vitamin K; wks. = weeks; Int. = intervention; Cont. = control; mg = milligram; UK = United Kingdom; US = United States.

### Risk of bias in included studies

**Figure 2:**
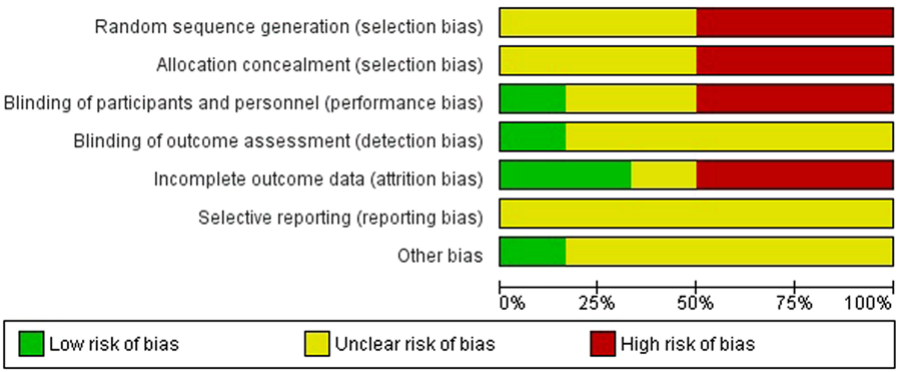
Risk of bias assessment graph on the effect of vitamin K supplementation during pregnancy.

**Figure 3:**
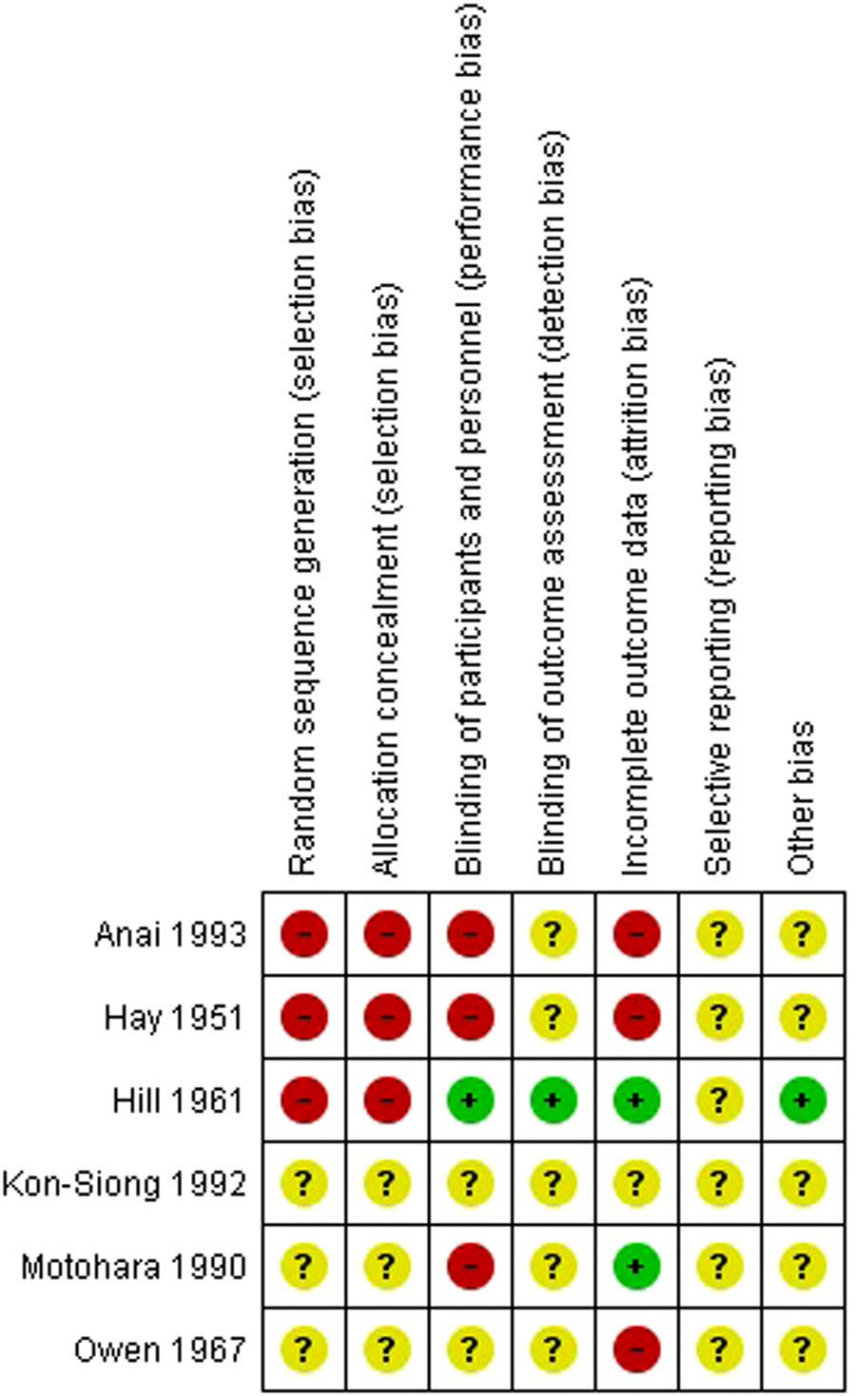
Overall risk of bias assessment on the effect of vitamin K supplementation during pregnancy.

We judged the included trials^36–41^ to be at high or unclear bias risk in overall. A summary risk of bias investigation is presented (Figs 2 and 3). See Supplementary Information for detailed RoB assessment.

### Intervention effects

The study of antenatal VK supplementation effect versus no VK or placebo was based on 21,493 pregnant women and newborns from six clinical trials (GRADE evidence summary: Table 2). Wherever possible, we meta-analysed data, presented summary effects, or findings as was reported in the trials.

**Table 2:** GRADE evidence summary.

**Vitamin K versus no vitamin K or placebo for improving outcomes**						
**Population**: women during pregnancy						
**Settings**: Liverpool, USA, and the Netherlands						
**Intervention**: vitamin K versus no vitamin K or placebo						
Outcomes	Anticipated absolute effects* **(95% CI)**		Relative effect (95% CI)	№ of participants (studies)	Quality of the evidence (GRADE)	Comments
	**Risk with no vitamin K or placebo**	**Risk with Vitamin K**				
Perinatal death	See comment	See comment	Not estimable	—	See comment	The outcome was not reported in any trials.
Neonatal bleeding	2 per 1,000	**2 per 1,000** (1 to 5)	**RR 1.16** (0.59 to 2.29)	17503 (4 RCTs)	⊕○○○ VERY LOW^a,b^	
Maternal bleeding	See comment	See comment	Not estimable	—	See comment	The outcome was not reported in any trials.
Neonatal death	See comment	See comment	Not estimable	—	See comment	The outcome was not reported in any trials.
Preterm birth	See comment	See comment	Not estimable	—	See comment	The outcome was not reported in any trials.
Neonatal jaundice	49 per 1,000	**72 per 1,000** (36 to 142)	**RR 1.47** (0.74 to 2.91)	533 (1 RCT)	⊕○○○ VERY LOW^b,c^	
Maternal plasma vitamin K1	The mean maternal plasma vitamin K1 was **0**	The mean maternal plasma vitamin K1 in the intervention group was 2.46 higher (0.98 higher to 3.93 higher)	—	65 (2 RCTs)	⊕○○○ VERY LOW^d,e^	
*The risk in the intervention group (and its 95% confidence interval) is based on the assumed risk in the comparison group and the **relative effect** of the intervention (and its 95% CI).						
**CI**: Confidence interval; **RR**: Risk ratio						
**GRADE Working Group grades of evidence**						
**High quality**: We are very confident that the true effect lies close to that of the estimate of the effect						
**Moderate quality**: We are moderately confident in the effect estimate: The true effect is likely to be close to the estimate of the effect, but there is a possibility that it is substantially different						
**Low quality**: Our confidence in the effect estimate is limited: The true effect may be substantially different from the estimate of the effect						
**Very low quality**: We have very little confidence in the effect estimate: The true effect is likely to be substantially different from the estimate of effect						

Explanations.

^a^Both included trials are quasi design and hence suffer from high bias risk for selection of the participants (−2).

^b^Wide 95%CI.

^c^One quasi RCT with high bias risk for selection of the participants (−2).

^d^Most of domains are unclear risk of bias (−1).

^e^Wide 95%CI and small sample size (−2).

#### Neonatal bleeding

**Figure 4:**
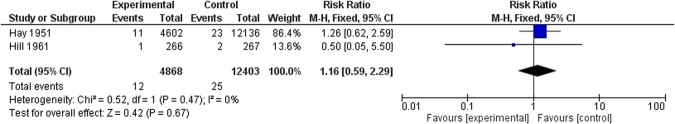
Comparison: 1 Vitamin K supplementation compared to no vitamin K or placebo, outcome: 1.1 Neonatal bleeding.

Two Trials^37,38^ contributed data for meta-analysis, where antenatal VK was found not statistically significant: total 17,271 babies (4,868 [intervention] vs. 12,403 [control]; risk ratio (RR) 1.16, 95% confidence interval (CI) 0.59 to 2.29; *P* = 0.67); heterogeneity Chi^2^ test = 0.52, df = 1 (*P* = 0.47); I^2^ = 0% (Fig. 4). There was no evidence of neonatal bleeding in two other trials^39,40^.

### Secondary outcomes (newborns)

#### Low birthweight (<2500 gm)

The reported LBW in neonates with jaundice^38^ was: 3/19 with VK and 0/13 without VK: RR 4.90 (95% CI 0.27 to 87.59; 32 neonates; *P* = 0.28) (analysis not shown). Birthweight were 2,860 to 3,420 gm^39^. No significant group difference in mean birthweight was found^36^: 3258 ± 399 (intervention N = 74) vs. 3160 ± 364 (N = 186). Birthweight ranged from 1445.8 to 3997.3 gm (intervention) vs. 1984.5 to 3940.6 gm (control)^37^.

#### Low Apgar score at five minutes

Healthy Apgar scores were reported by three studies: from 9 to 10^39^ and 8 or higher^36^. Since the measurement time was not specified by these two trials, data could not be pooled together. Apgar scores ≥8 at 1 minute were reported in one^41^.

#### Hypoprothrombinemia

Protein-induced VK absence-II (PIVKA-II) or hypoprothrombinemia, a sensitive marker of VK deficiency, was measured^39^: a significantly less number of PIVKA-II newborns in both VK1 (n = 11) and K2 (n = 12) treated groups were found vs. the controls (n = 10); *P* = <0.05. Positive PIVKA-II was reported in 9/10 controls. These individual data were pointed as “not detectable.”

### Non-prespecified outcomes (newborns)

#### Noncarboxylated prothrombin

Noncarboxylated prothrombin (a prothrombin form that is usually found in VK deficient population or those on VK antagonist drugs)^36^: positive prothrombin (>1 µg/mL) was detected in 1.4% (of the VK group (1/74) vs. 7.0% (13/186): RR 0.19, 95% CI 0.03 to 1.45; *P* = 0.11, not statistically significant (analysis not shown).

#### Normotest value

In neonates, improved activation of VK-dependent coagulant factors was detected^36^: 59.6 ± 10.1% (intervention, N = 74) vs. 53.4 ± 9.9% (N = 186) with a large significant group difference; MD 6.20, 95% CI 3.49 to 8.91; *P* = <0.00001 (analysis not shown).

#### Neonatal plasma vitamin K

Vitamin K1 treated group found significantly different than their controls^39^: MD 1.02, 95% CI 0.12 to 1.92; *p* = 0.03; the VK2 concentrations were indifferent: MD 0.12, 95% CI −0.23 to 0.47; *P* = 0.50 (analysis not shown).

#### Neonatal cord serum vitamin K

Neonatal cord sera^41^ showed significant group differences (*P* = <0.01) for VK1 (pg/ml): mean ± SE 43.2 ± 2.2 (intervention) vs. < 20 (placebo). Cord plasma intervention samples^39^ had significantly higher VK1 and VK2 (*P* = <0.05 and <0.01, respectively).

#### Jaundice

Hyperbilirubinemia of “undetermined etiology” outcome showed no significant group difference (19/266 treatment and 13/267 control newborns)^38^: RR 1.47, 95% CI 0.74 to 2.91; *P* = 0.27; N = 533 (analysis not shown). The mean peak bilirubin was: (17.6 mg%; 7.2% babies, VK arm) vs. 14.5 mg% (4.8%). Eight of the treated newborns were detected with high (≥20 mg%) bilirubin level. Of these newborns, 2/266 were reported as “premature by weight.” Only 1/267 baby (placebo arm) was identified with high bilirubin 20.7 mg%. Neonates were assessed in two different years^40^: no group difference was found for total bilirubin, mg/100 ml (first 30 hours of life): mean ± SD 1.5 ± 1.1 vs. 1.2 ± 1.1 (1965); 1.8 ± 1.4 vs. 1.9 ± 1.2 (1966).

#### Osteocalcin (irOC) and hydroxylapatite binding capacity (HBC)

Cord serum irOC (mg/mL) showed no significant intervention effect^41^: mean ± SE 6.31 ± 0.49 (intervention) vs. 6.61 ± 0.57; MD −0.30, 95% CI −1.77 to 1.17; *P* = 0.69 (analysis not shown). For cord serum HBC (% absorbed), the effect reached a statistical significance: 53.2 ± 2.20 vs. 35.6 ± 2.70 (placebo); MD 17.60, 95% CI 10.77 to 24.43; *P* = 0.00001 (analysis not shown).

#### Two-stage prothrombin

Neonates’ average two-stage prothrombin (% of the control determination, first 30 hours of life)^40^, (1965): 50.8 ± 10.0 (N = 51) vs. 44.3 ± 12.8 (placebo, N = 66), *P* = <0005; 39.9 ± 7.7 (N = 44) vs. 36.7 ± 8.9 (placebo, N = 41, 1966), 0.1 > *P* > 0.05. Prothrombin% (one-stage/two-stage unspecified) was assessed by another trial^38^: among the 3 babies (N = 533; intervention 266 vs. 267) showing bleeding signs after birth, one baby (VK injection arm) in day one was measured with 68% prothrombin; two babies (placebo) bled in day two, one of which had prothrombin <7%, and 5% (the other). See Supplementary Information for more.

#### Adverse outcomes

Newborns (5/79 treated; 4/190 controls) were reported for admission to the NICU for LBW, asphyxiation, hyperbilirubinaemia, or heart murmurs^36^; however, they were not separated by diagnosis. Three newborns were reported to have different conditions: septicemia and HDN (placebo arm), and bleeding dyscrasia (treatment arm)^38^. Eight babies were diagnosed with severe jaundice symptom (≥20 mg% bilirubin) and were at risk of Kernicterus development (VK arm)^38^. One trial reported finding no evidence of side effects for VK administration^40^.

### Secondary outcomes (mothers)

#### Hypoprothrombinemia

Oral VK1 and K2 supplementation effect on vitamin K dependent factor PIVKA II was assessed for women^39^, but the data range weren’t detectable (original study, Tables 1 and 2).

### Non-prespecified outcomes (maternal)

#### Maternal plasma vitamin K

**Figure 5:**
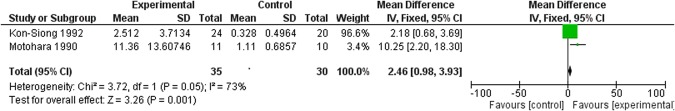
Comparison: 1 Vitamin K supplementation compared to no vitamin K or placebo, outcome: 1.2 Maternal plasma vitamin K.

We converted data from pg/ml to ng/ml unit and pooled two trials^39,41^ together (35 [intervention] vs. 30 [control]: MD 2.46, 95% CI 0.98 to 3.93; *P* = 0.001; heterogeneity Chi^2^ = 3.72, df = 1 (P = 0.05); I^2^ = 73% (Fig. 5). Both of the trials showed significance of antenatal VK1 compared to their controls: *P* = <0.01^39^; *P* = <0.03^41^. Maternal plasma VK2: mean ± SD 3.8 ± 4.85 vs. 0.17 ± 0.20; MD 3.63, 95% CI 0.88 to 6.38; *P* = 0.010 (analysis not shown).

#### Breast milk vitamin K

Breast milk VK1 and K2 (ng/ml) were significantly higher in the treated mothers (5^th^ postpartum day)^39^: 3.32 ± 2.06 vs. 1.20 ± 0.34; MD 2.12, 95% CI 0.88 to 3.36; *P* = 0.0008 (K1); 6.93 ± 4.41 vs. 0.77 ± 0.26; MD 6.16, 95% CI 3.66 to 8.66; *P* = 0.00001 (K2) (analysis not shown).

#### Osteocalcin (irOC) and hydroxylapatite binding capacity (HBC)

Maternal irOC values (ng/ml)^41^: mean ± SE 2.38 ± 0.26 (VK1) vs. 2.04 ± 0.25 (placebo) were indifferent for groups; MD 0.34, 95% CI −0.38 to 1.06 (analysis not shown). The group difference was significant for HBC (% absorbed): 76.6 ± 1.9 vs. 65.5 ± 3.7 (placebo); *P* = <0.02; MD 11.10, 95% CI 2.95 to 19.25; *P* = 0.008 (analysis not shown).

## Discussion

According to this review, VK supplementation showed no benefits for the reduction of neonatal bleeding but for maternal plasma vitamin K1 status. Antenatal VK exerted beneficial effects for numerous other outcomes e.g. cord serum VK and HBC (mother-newborn), maternal-newborn VK-dependent factors, breast milk VK. Limited comments^38,40,42^ on VK induced adverse outcomes were presented by authors.

### Overall comprehensiveness and relevance of evidence

Our findings are largely dependent on two trials and the pooled estimates were dominated by single large studies. None of the included trials assessed perinatal death or maternal bleeding, including a set of secondary outcomes. Most of the trials were quite old, i.e. between years 1951 and 1993 and included women at varying gestational ages, mostly at >20 weeks. The interventions ran from minimum half an hour up to 2 weeks prior delivery and used both oral and intramuscular route. Oral route was commonly used. Different vitamin forms i.e. K, K1, and K2 were used and varied in dosages e.g. 1 mg daily were more common. All the vitamin forms and routes detected some level of significance for some of the reported outcomes. Therefore, it is difficult to precisely suggest which particular VK form, administration route, dosage, and intervention duration is most beneficial for women at a particular gestational age.

We could only meta-analyse data for neonatal bleeding and maternal plasma VK1. We would pool data^39,41^ also for neonatal cord sera; however VK1 levels were undetectable in the placebo arm (<20 pg/ml)^41^. We did not detect any significant effect of VK administration on the reduction of neonatal bleeding (Fig. 4, meta-analysis). In one trial^37^, groups were limited in comparability because of the wide variability in HDN incidence (bleeding from the stomach or the bowel was recorded only and IH was not considered); consequently, authors were unsure whether the findings were due to prothrombin deficiency or other undetermined reasons. Additionally, VK was not received by “many” (not specified) of the treated mothers before delivery, as a result, hurried labour prior to or right after hospitalization were documented by the authors. Maternal plasma VK1 was significantly improved in two small sized RCTs^39,41^ (Fig. 5).

Although both the trials detected a significant intervention effect, VK1 levels in the treated mothers were much lower (2.51 ± 37.43 ng/ml)^41^ compared to the treated mothers (11.36 ± 13.60 ng/ml) in another study^39^. The lower values could be due to varying dose administration i.e. VK1 (Konakion®, 1 mg/day) orally until delivery^41^ vs. oral VK1 (20 mg/day, Eisai Ltd., Japan) once only for 7 to 10 consecutive mornings until birth^39^. A significant correlation in VK1 concentrations between the maternal and cord sera was reported^39^, with a large gradient (mostly < one-tenth). Very low VK1 in the treated cord sera was also found^41^ i.e. 2.5 times below the lower normal adult range, 60 times below the corresponding maternal values. Although high VK1 in maternal sera was found, it remained undetectable (<20 pg/ml) for the placebo mothers and cord samples, for which authors concluded that the placental barrier is truly a large factor for vitamin K transportation to the newborns. For VK1 and VK2, a significantly higher concentration in the treated maternal and cord sera (VK1 only in neonatal cord sera) were reported than controls^39^. Vitamin K1 showed statistical significance for elevating VK-dependent coagulant factors for the intervention babies^36^, where neonates (5 treated, 4 controls) were excluded for NICU admission due to LBW and other diagnosis e.g. hyperbilirubinemia. Both trials concluded that though in lower values, VK1 and VK2 crossed the placenta and continued activating the VK-dependent coagulant factors (minimum until day 5), which increased neonatal VK status, supported by another included trial^39^. Although the treated cord serum HBC (% absorbed)^41^ was significantly higher i.e. 53.2 ± 2.20 vs. 35.6 ± 2.70 (placebo); *P* = 0.00001, the values were markedly lower in all cord samples compared to the mothers, which further indicated newborns usual VK deficiency at birth due to the large placental block. The extremely suboptimal HBC values in all control newborns were comparable to the population who generally use oral anticoagulants^39^. Newborns’ immature carboxylase system has less capacity to maintain the creation of complete carboxylated osteocalcin and was indicated among the underlying mechanisms for suboptimal response of fetal osteocalcin to maternal VK consumption, further supporting the need for higher VK supplementation for fetal and neonatal development. Although VK1 benefitted newborns’ usual prothrombin (two-stage, 55 hours of life) shortage, bilirubin was indifferent for groups and, due to various reasons, some 50% of the babies could not be considered in analysis^40^. The HBC (% absorbed) in the treated mothers was also significantly higher^41^; however, in 11/20 control mothers, the values found lower than the normal adult range, for which some of the mothers were thought to have subclinical VK deficiency. In addition, the mothers and newborns’ HBC in both groups were significantly correlated (r = +0.59; *P* = <0.0003), supporting the fact that maternal optimal VK boosts newborns’ VK. Furthermore, breast milk from treated mothers showed significantly higher levels of vitamin K1 and K2 levels^39^, with these two groups showing a marked correlation. As a result, greater VK storage in maternal liver was suggested to be beneficial, which is transferred to the breast milk from maternal liver and consequently, help improve newborns’ VK deposit.

### Evidence quality

Overall, the trials were judged either as methodologically unclear or at high bias risk. The merit of the evidence (GRADE summary of findings Table 2) was very low for neonatal bleeding and neonatal jaundice, which both ranked two levels lower mainly due to high bias risk for participant selection technique in quasi RCTs and for wide 95% CI. Maternal sera VK1 was downgraded by two levels, because risk of bias domains were mostly unclear with wide 95% CI, including small sample size.

### Present study findings in light of existing literature

According to the present study, the effectiveness of antenatal VK was not statistically significant for neonatal bleeding, except for maternal sera VK1. In cases other than frequent liver injury, neonatal bleeding including hypoprothrombinaemic condition was previously improved^43^. At delivery, maternal plasma VK was significantly higher, and since women’s VK levels normally drop during the last trimester (e.g. 40 to 41 gestational weeks)^39^, VK supplement was suggested to improve maternal osteocalcin carboxylation, including newborns. Antenatal VK showed benefits for other outcomes. Consistent with previous studies^40,44–46^, a large significant effect of oral VK1 for improved blood coagulation factors was detected in mother-newborn pair^36,39^, including cord blood VK^39^ and neonatal serum VK^41^. However, possibility of VK dilution in the breast milk of the treated women or VK storage in the newborns liver were indicated by the authors as influencing factors. Neonatal VK deficiency at birth was pointed out as a common phenomenon due to the well-established fact of obstructed placental transfer of VK to the newborns^11^, including low HBC values which possibly appear from poor VK deposit in the newborns^41^. Ensuring an optimal deposit of maternal VK was suggested as a plausible remedy by the trialists to improve newborns’ VK status. Nonetheless, administration of VK previously did not show marked benefit for the newborns with declining VK deficiency^12^. Furthermore, significantly elevated maternal breast milk VK1 for VK1 supplementation was observed at delivery^39^, consistent with previous research reporting a 7-fold (after 12 hours) and 2-fold (after 48 hours) increased VK1 in the breast milk for a single oral VK1 dose^47^. Breast-fed newborns who are non-supplemented with VK may develop severe bleeding complication^48^; however, none of our included trials provided such assessment. Newborns are required to be supplemented because mothers carry extremely low amount of breast milk VK; therefore, greater maternal VK intake has been suggested to significantly enrich breast milk nutrients for offspring’s optimal nutrient supply.

### Limitations

Our review evidence is weak on the following points. First, our review is limited to 6 trials which were quite old; and no new eligible trials were identified through our update search. Second, an equal number of papers (3 each) contributed data from RCT and quasi-RCT and therefore, design-wise, the strength of the evidence is not entirely strong. Third, most of the trials (4/6) included small samples and short follow-up time (during labour to 6 weeks prior delivery). Fourth, the available evidence is only from trials conducted in high-income countries and cannot be generalized globally. The only trial identified from a low-income country i.e. South Africa; (quasi-RCT), did not qualify for inclusion in this review. Fourth, information in the reports was often unclear, limited or unavailable. Finally, despite applying an exhaustive search method, it is possible there were additional trials (published or unpublished) in this field that we could have included.

## Conclusions

Based on this review evidence, the effect of antenatal VK was not statistically significant for neonatal bleeding reduction except for maternal plasma VK1, including other outcomes e.g. neonatal plasma VK, maternal-newborn PIVKA-II factor and breast milk VK. This review was mainly driven by the need for compilation of systematic evidence on VK deficiency during pregnancy, its beneficial dosages and related morbidities in healthy women including those with epilepsy, malabsorption and other health conditions, as these evidence to date is insufficient. Although VK supplement is not necessary in normal pregnancy, deficiency may occur^1,6^ in epilepsy and other impaired conditions^5,10^. We planned to assess these important women subgroups^33^ however, none of the included trials provided such observations but either included mostly healthy women with uncomplicated pregnancy or excluded critical cases from the analyses, e.g. stillbirths, congenital abnormalities and LBW babies^40^. We believe that closing these knowledge gaps is particularly essential because epileptic pregnant women tend to experience troubled metabolism or have higher chances of giving birth to newborns with early bleeding and neural tube defects^32,33^. Furthermore, during the last century, the human diet has changed substantially, resulting in extremely low levels of VK deposit in human body^49^. Research cautioned of experiencing adverse outcomes if women’s diet was altered during pregnancy^29,30,45^. Moreover, outcomes like neonatal bleeding and neonatal death are rare in high-income settings and could be difficult to detect due to various underlying factors such as healthcare seeking behaviour, socio-economic background, diet availability, and seasonal food variation^42^. It is essential that these factors are discussed in future studies as these might influence the intervention effect, especially in certain minority and low-income women. Future studies should assess outcomes that were not investigated or reported inadequately e.g. maternal bleeding and adverse outcomes, preferably using long follow-up, large sample size, and inclusion of critical subgroups.

## Electronic supplementary material

Supplementary Information
