# Supplementary material for: Vitamin K supplementation during pregnancy for improving outcomes: a systematic review and meta-analysis
Source: Sci Rep. 2018 Jul 30;8:11459. doi: 10.1038/s41598-018-29616-y (PMC6065418; doi:10.1038/s41598-018-29616-y)
Supplement: Supplementary file 1 — Supplementary Information [file 41598_2018_29616_MOESM1_ESM.doc]

**Supplementary Information**

**Title: Vitamin K supplementation during pregnancy for improving outcomes: a systematic review and meta-analysis**

**Authors:**

Sadequa Shahrook^1^, Erika Ota^2^, Nobutsugu Hanada^3^, Kimi Sawada^4^, Rintaro Mori^3^

^1^Present: Population Health Research Institute, A Joint Institute of McMaster University and Hamilton Health Sciences, Hamilton, Ontario, Canada

^1^Past: Department of Health Policy, National Center for Child Health and Development, Tokyo, Japan
^2^Global Health Nursing, St. Luke's International University, Graduate School of Nursing Sciences, Tokyo, Japan

^3^Department of Health Policy, National Center for Child Health and Development, Tokyo, Japan

^4^Department of Food Science and Nutrition Faculty of Human Life and Environmental Sciences, Nagoya Women's University, Aichi, Japan
***Corresponding author:**

Sadequa Shahrook, Population Health Research Institute, A Joint Institute of McMaster University and Hamilton Health Sciences, David Braley Cardiac, Vascular and Stroke Research Institute (DBCVSRI), 20 Copeland Avenue, Hamilton, ON L8L 0A3, Canada.

Tel: 905-521-2100 ext. 40751. Fax: 905-297-3782. [Sadequa.Shahrook@phri.ca](mailto:Sadequa.Shahrook@phri.ca)

PRISMA checklist 2009

| **Section/topic** | **#** | **Checklist item** | **Reported on page #** |
| --- | --- | --- | --- |
| **TITLE** | | |  |
| Title | 1 | Identify the report as a systematic review, meta-analysis, or both. | 1 |
| **ABSTRACT** | | |  |
| Structured summary | 2 | Provide a structured summary including, as applicable: background; objectives; data sources; study eligibility criteria, participants, and interventions; study appraisal and synthesis methods; results; limitations; conclusions and implications of key findings; systematic review registration number. | 2 |
| **INTRODUCTION** | | |  |
| Rationale | 3 | Describe the rationale for the review in the context of what is already known. | 3-4 |
| Objectives | 4 | Provide an explicit statement of questions being addressed with reference to participants, interventions, comparisons, outcomes, and study design (PICOS). | 4-5 |
| **METHODS** | | |  |
| Protocol and registration | 5 | Indicate if a review protocol exists, if and where it can be accessed (e.g., Web address), and, if available, provide registration information including registration number. | 5 |
| Eligibility criteria | 6 | Specify study characteristics (e.g., PICOS, length of follow-up) and report characteristics (e.g., years considered, language, publication status) used as criteria for eligibility, giving rationale. | 5-6 |
| Information sources | 7 | Describe all information sources (e.g., databases with dates of coverage, contact with study authors to identify additional studies) in the search and date last searched. | 5 |
| Search | 8 | Present full electronic search strategy for at least one database, including any limits used, such that it could be repeated. | 4-7 |
| Study selection | 9 | State the process for selecting studies (i.e., screening, eligibility, included in systematic review, and, if applicable, included in the meta-analysis). | 6 |
| Data collection process | 10 | Describe method of data extraction from reports (e.g., piloted forms, independently, in duplicate) and any processes for obtaining and confirming data from investigators. | 6 |
| Data items | 11 | List and define all variables for which data were sought (e.g., PICOS, funding sources) and any assumptions and simplifications made. | 6 |
| Risk of bias in individual studies | 12 | Describe methods used for assessing risk of bias of individual studies (including specification of whether this was done at the study or outcome level), and how this information is to be used in any data synthesis. | 6 |
| Summary measures | 13 | State the principal summary measures (e.g., risk ratio, difference in means). | 7 |
| Synthesis of results | 14 | Describe the methods of handling data and combining results of studies, if done, including measures of consistency (e.g., I^2^) for each meta-analysis. | 6-7 |

Page 1 of 2

| **Section/topic** | **#** | **Checklist item** | **Reported on page #** |
| --- | --- | --- | --- |
| Risk of bias across studies | 15 | Specify any assessment of risk of bias that may affect the cumulative evidence (e.g., publication bias, selective reporting within studies). | 6 |
| Additional analyses | 16 | Describe methods of additional analyses (e.g., sensitivity or subgroup analyses, meta-regression), if done, indicating which were pre-specified. | 6 |
| **RESULTS** | | |  |
| Study selection | 17 | Give numbers of studies screened, assessed for eligibility, and included in the review, with reasons for exclusions at each stage, ideally with a flow diagram. | 7 |
| Study characteristics | 18 | For each study, present characteristics for which data were extracted (e.g., study size, PICOS, follow-up period) and provide the citations. | 7 |
| Risk of bias within studies | 19 | Present data on risk of bias of each study and, if available, any outcome level assessment (see item 12). | 8 |
| Results of individual studies | 20 | For all outcomes considered (benefits or harms), present, for each study: (a) simple summary data for each intervention group (b) effect estimates and confidence intervals, ideally with a forest plot. | 8-10, tab 3, Fig 4, Fig 5, Supplementary Information |
| Synthesis of results | 21 | Present the main results of the review. If meta-analyses are done, include for each, confidence intervals and measures of consistency. | 8-10, tab 3, Fig 4, Fig 5 |
| Risk of bias across studies | 22 | Present results of any assessment of risk of bias across studies (see Item 15). | 12 |
| Additional analysis | 23 | Give results of additional analyses, if done (e.g., sensitivity or subgroup analyses, meta-regression [see Item 16]). | NA |
| **DISCUSSION** | | |  |
| Summary of evidence | 24 | Summarize the main findings including the strength of evidence for each main outcome; consider their relevance to key groups (e.g., healthcare providers, users, and policy makers). | 10 |
| Limitations | 25 | Discuss limitations at study and outcome level (e.g., risk of bias), and at review-level (e.g., incomplete retrieval of identified research, reporting bias). | 13-14 |
| Conclusions | 26 | Provide a general interpretation of the results in the context of other evidence, and implications for future research. | 13, 14 |
| **FUNDING** | | |  |
| Funding | 27 | Describe sources of funding for the systematic review and other support (e.g., supply of data); role of funders for the systematic review. | 21 (Tab 1) |

*From:*  Moher D, Liberati A, Tetzlaff J, Altman DG, The PRISMA Group (2009). Preferred Reporting Items for Systematic Reviews and Meta-Analyses: The PRISMA Statement. PLoS Med 6(7): e1000097. doi:10.1371/journal.pmed1000097

**Search strategies**

Search date: February 28, 2018

Cochrane Library

#1 MeSH descriptor: [Vitamin K] explode all trees[492]

#2 vitamin* near/4 k*:ti,ab,kw (Word variations have been searched)[19716]

#3 green near/2 vegetable*:ti,ab,kw (Word variations have been searched)[93]

#4 (phytomenadione or phytonadione or konakion or phylloquinone or Phylloquinine or phyllohydroquinone or aquamephyton or menaquinone* or menadione or vi?asol or "2-methyl-14-naphthoquinone" or "2-methylnaphthoquinone" or "2-methyl-14-naphthalenedione" or acetomenaphthone or farnoquinone or menadiol or menatetrenone):ti,ab,kw (Word variations have been searched)[271]

#5 #1 or #2 or #3 or #4 [19802]

#6 MeSH descriptor: [Pregnancy] explode all trees[5977]

#7 MeSH descriptor: [Pregnancy Complications] explode all trees[9597]

#8 MeSH descriptor: [Fetal Therapies] explode all trees[45]

#9 MeSH descriptor: [Labor Pain] explode all trees[201]

#10 MeSH descriptor: [Infant, Newborn] explode all trees[15720]

#11 MeSH descriptor: [Fetus] explode all trees[1758]

#12 MeSH descriptor: [Fetal Development] explode all trees[2388]

#13 MeSH descriptor: [Extraembryonic Membranes] explode all trees[368]

#14 MeSH descriptor: [Heart Rate, Fetal] explode all trees[339]

#15 MeSH descriptor: [Placenta] explode all trees[355]

#16 MeSH descriptor: [Placental Function Tests] explode all trees[4]

#17 MeSH descriptor: [Umbilical Cord] explode all trees[550]

#18 MeSH descriptor: [Prenatal Diagnosis] explode all trees[1104]

#19 MeSH descriptor: [Uterine Monitoring] explode all trees[21]

#20 MeSH descriptor: [Pelvimetry] explode all trees[13]

#21 MeSH descriptor: [Fetal Monitoring] explode all trees[385]

#22 MeSH descriptor: [Obstetric Nursing] explode all trees[44]

#23 MeSH descriptor: [Oxytocics] explode all trees[878]

#24 MeSH descriptor: [Tocolytic Agents] explode all trees[286]

#25 MeSH descriptor: [Tocolysis] explode all trees[118]

#26 MeSH descriptor: [Anesthesia] explode all trees[18077]

#27 MeSH descriptor: [Obstetric Surgical Procedures] explode all trees[6959]

#28 MeSH descriptor: [Maternal Health Services] explode all trees[2141]

#29 MeSH descriptor: [Maternal-Child Nursing] explode all trees[211]

#30 MeSH descriptor: [Analgesia, Obstetrical] explode all trees[932]

#31 MeSH descriptor: [Midwifery] explode all trees[339]

#32 MeSH descriptor: [Perinatal Care] explode all trees[573]

#33 MeSH descriptor: [Parity] explode all trees[840]

#34 MeSH descriptor: [Apgar Score] explode all trees[671]

#35 MeSH descriptor: [Postpartum Period] explode all trees[1447]

#36 MeSH descriptor: [Breast Feeding] explode all trees[1728]

#37 MeSH descriptor: [Milk, Human] explode all trees[922]

#38 pregnan*:ti,ab,kw (Word variations have been searched)[37794]

#39 fetus:ti,ab,kw (Word variations have been searched)[4150]

#40 foetus:ti,ab,kw (Word variations have been searched)[126]

#41 fetal:ti,ab,kw (Word variations have been searched)[7766]

#42 foetal:ti,ab,kw (Word variations have been searched)[376]

#43 newborn:ti,ab,kw (Word variations have been searched)[23061]

#44 "new born":ti,ab,kw (Word variations have been searched)[78]

#45 birth or childbirth:ti,ab,kw (Word variations have been searched)[18173]

#46 labor or laboring:ti,ab,kw (Word variations have been searched)[10694]

#47 labour*:ti,ab,kw (Word variations have been searched)[3852]

#48 antepart*:ti,ab,kw (Word variations have been searched)[344]

#49 prenatal*:ti,ab,kw (Word variations have been searched)[5090]

#50 antenatal*:ti,ab,kw (Word variations have been searched)[2718]

#51 perinatal*:ti,ab,kw (Word variations have been searched)[3872]

#52 postnatal*:ti,ab,kw (Word variations have been searched)[2945]

#53 postpart*:ti,ab,kw (Word variations have been searched)[5343]

#54 caesar*:ti,ab,kw (Word variations have been searched)[3111]

#55 cesar*:ti,ab,kw (Word variations have been searched)[7227]

#56 obstetric*:ti,ab,kw (Word variations have been searched)[10823]

#57 oxytoci*:ti,ab,kw (Word variations have been searched)[3415]

#58 tocoly*:ti,ab,kw (Word variations have been searched)[702]

#59 placenta*:ti,ab,kw (Word variations have been searched)[2465]

#60 prostaglandin:ti,ab,kw (Word variations have been searched)[5986]

#61 parturi*:ti,ab,kw (Word variations have been searched)[1889]

#62 preeclamp*:ti,ab,kw (Word variations have been searched)[1375]

#63 pre next eclamp*:ti,ab,kw (Word variations have been searched)[1160]

#64 eclamp*:ti,ab,kw (Word variations have been searched)[1345]

#65 intrapart*:ti,ab,kw (Word variations have been searched)[768]

#66 puerper*:ti,ab,kw (Word variations have been searched)[1782]

#67 episiotom*:ti,ab,kw (Word variations have been searched)[630]

#68 amnio*:ti,ab,kw (Word variations have been searched)[1726]

#69 matern*:ti,ab,kw (Word variations have been searched)[14403]

#70 gestation*:ti,ab,kw (Word variations have been searched)[14411]

#71 lactati*:ti,ab,kw (Word variations have been searched)[1615]

#72 breastfe*:ti,ab,kw (Word variations have been searched)[2518]

#73 breast next fe*:ti,ab,kw (Word variations have been searched)[3279]

#74 #6 or #7 or #8 or #9 or #10 or #11 or #12 or #13 or #14 or #15 or #16 or #17 or #18 or #19 or #20 or #21 or #22 or #22 or #23 or #24 or #25 or #26 or #27 or #28 or #29 or #30 or #31 or #32 or #33 or #34 or #35 or #36 or #37 or #38 or #39 or #40 or #41 or #42 or #43 or #44 or #45 or #46 or #47 or #48 or #49 or #50 or #51 or #52 or #53 or #54 or #55 or #56 or #57 or #58 or #59 or #60 or #61 or #62 or #63 or #64 or #65 or #66 or #67 or #68 or #69 or #70 or #71 or #72 or #73 [95043]

#75 #5 and #74 [2459] limited to year published 2016-2018 [430]

**Risk of bias in included studies**

The included trials^36-41^were judged to be of unclear to high risk of bias in overall. A summary risk of bias assessment is provided in [Figure 2](../../../../Ota%20Sense/Vitamin%20K%20supplementation%20during%20pregnancy%20for%20improving%20outcomes.htm#FIG-02); [Figure 3](../../../../Ota%20Sense/Vitamin%20K%20supplementation%20during%20pregnancy%20for%20improving%20outcomes.htm#FIG-03). We assigned a high risk for sequence generation^36-38^ because of alternate allocation method. We observed unclear bias risk^39-41^where randomisation method was not described. We observed high allocation concealment bias due to alternate allocation method^36-38^. We observed unclear risk^39-41^ as this was not reported adequately. For performance bias (blinding of participants and clinicians), studies were at high risk^36,37,39^,, low risk^38^,  and unclear risk^40, 41^. Detection bias (blinding of outcome assessors) was unclear for five trials^36,37,39-41^, but low for one^38^. We observed high risk for incomplete outcome data^36,37,40^ primarily due to exclusions of the participants for inadequate or no treatment with VK, or unusable blood samples. Many infants were also excluded for NICU admission, LBW and other morbidities. Low risk identified in two^38,39^. Post-randomisation exclusion of participants and loss to follow-up at each data collection point did not seem to happen^39^, but babies with haematological explanation for the icterus (major or minor blood incompatibility) were eliminated (21 cases; 3.9%) after randomisation and birth^38^. There was unclear risk^41^ as no information on post-randomisation exclusion, withdrawals, and loss to follow-up were provided, including outcomes for the babies without neonatal jaundice. The risk of reporting bias for all included trials is unclear and without access to the study protocols, we could not determine whether outcome data for all pre-specified outcomes were reported. We found low risk for baseline imbalance^38^ as the groups were comparable from an obstetric viewpoint, e.g. age, labour duration and parity: intervention 266 vs. control 267. We assessed the rest of the trials for unclear bias risk. We reported no significant group differences for participants' mean age and clinical characteristics^41^ and fairly equal intervention/control participants: 24 vs. 20. There were unequal groups^36^: 74 vs. 186; baseline imbalance reported as non-significant for maternal age, parity, gestational age, and birthweight. Groups were also largely unequal^37^: 4602 vs. 12136; baseline imbalance was not reported. Similarly, baseline imbalance was not reported^39^ but reported fairly equal group participants: 11, 12 vs. 10. [Results](../../../../Ota%20Sense/Vitamin%20K%20supplementation%20during%20pregnancy%20for%20improving%20outcomes.htm#STD-Owen-1967) were collected in two different years for reasonably similar comparable newborn groups^40^. Early study termination was not mentioned by any of the included trials.

**Effects of interventions**

**One and two-stage prothrombin.** Neonates’ average one- and two-stage prothrombin (% of the control determination, first 30 and 55 hours of life)^40^: one-stage prothrombin (first 30 hours, 1965): mean±SD 98.1±11.5 (VK1, N = 51) vs. 93.7±17.2 (placebo), detected no significant intervention effect (0.1>*P*>0.05). There was a significant effect reported in 1966 observations: 100±9.0 (VK1, N = 45) vs. 91.4±13.7 (N = 39), *P*=<0.005. Two-stage prothrombin (1965): 50.8±10.0 (N = 51) vs. 44.3±12.8 (placebo, N = 66), *P=*<0005; and 39.9±7.7 (N = 44) vs. 36.7±8.9 (placebo, N = 41, 1966), 0.1>*P*>0.05. At 55 hours of life (available only for the year 1965), the values were reported as "virtually the same as those obtained earlier": one-stage prothrombin: 98.6±13.4 (VK1, N = 37) vs. 94.9±13.3 (N = 48); two-stage prothrombin: 50.7±12.9 vs. 43.4±11.8 (placebo). Authors also presented two-stage prothrombin at 0 to 15 hours and 15 to 30 hours of life but without a statistical significance (data not presented). Prothrombin percentages (one-stage or two-stage unspecified) were assessed in another trial^38^: among the 3 babies (N = 533; intervention 266 vs. 267) with bleeding signs following birth, one baby (VK injection) in day one was measured with 68% prothrombin; two babies (placebo) bled in day two, one of which had prothrombin <7%, and 5% (the other). Also, authors randomly chose 6 babies (three each from both study groups) as control and only one baby (placebo) was reported reaching a risky prothrombin level (i.e. 21%).
